# A Review of the Impact of the COVID-19 Pandemic on Colorectal Cancer Screening: Implications and Solutions

**DOI:** 10.3390/pathogens10111508

**Published:** 2021-11-19

**Authors:** Suneha Sundaram, Sean Olson, Paranjay Sharma, Shanmugarajah Rajendra

**Affiliations:** 1 Department of Medicine, Robert Wood Johnson University Hospital, Rahway, NJ 07065, USA; 2 Kansas City University, Kansas City, MO 64106, USA; solson@kansascity.edu; 3 Barstow School, Kansas City, MO 64114, USA; paranjay.sharma@barstowschool.org; 4 South Western Sydney Clinical School, University of New South Wales, Sydney, NSW 2052, Australia; Shan.Rajendra@health.nsw.gov.au; 5 Gastro-Intestinal Viral Oncology Group, Ingham Institute for Applied Medical Research, Sydney, NSW 2170, Australia; 6 Department of Gastroenterology & Hepatology, Bankstown-Lidcombe Hospital, South Western Sydney Local Health Network, Sydney, NSW 2200, Australia

**Keywords:** COVID-19, SARS-CoV-2, colorectal cancer, screening, colonoscopy, fecal immunochemical test

## Abstract

The COVID-19 pandemic has impacted all aspects of medical care, including cancer screening and preventative measures. Colorectal cancer screening declined significantly at the onset of the pandemic as the result of an intentional effort to conserve resources, prioritize emergencies and reduce risk of transmission. There has already been an increase in diagnosis at more advanced stages and symptomatic emergencies due to suspended screenings. As endoscopy units find their way back to pre-pandemic practices, a backlog of cases remains. The missed CRC diagnoses amongst the missed screenings carry a risk of increased morbidity and mortality which will only increase as time-to-diagnosis grows. This review discusses the impact of COVID-19 on colonoscopy screening rates, trends in stages/symptoms/circumstances at diagnosis, and economic and social impact of delayed diagnosis. Triaging and use of FITs are proposed solutions to the challenge of catching up with the large number of pandemic-driven missed CRC screenings.

## 1. Introduction

The novel severe acute respiratory syndrome coronavirus-2 (SARS-CoV-2) that has caused the COVID-19 pandemic is, like the previous coronaviruses and the pandemics of SARS in 2002 and Middle East Respiratory Syndrome (MERS) in 2012, a single-stranded RNA virus that originated from a zoonotic host and causes severe lower respiratory tract illness with poor mortality outcomes [1,2]. While most patients present with respiratory symptoms, many patients also have gastrointestinal (GI) symptoms. Additionally, COVID-19 patients with and without GI symptoms have shown detectable viral loads in fecal samples [3,4,5,6,7]. Given the high transmissibility of COVID-19, the first and most important international public health task has been outbreak control, identifying the infected and employing practices to limit spread to uninfected individuals [1,2,8]. As a result, healthcare resources were immediately diverted to those with the greatest need, i.e., critically ill patients, and non-critical and preventative medical interventions were held across all specialties.

We performed a PubMed literature search of keywords including “COVID,” “colorectal cancer,” “screening,” “colon cancer,” and “colonoscopy” to find articles that could further elucidate the impact that the pandemic has had on colorectal cancer (CRC) screenings and diagnoses numbers as well as the influence this shift may have on patients, including staging and life years lost. This paper provides a review of these findings. We included studies that published both quantitative and qualitative data from international populations in order to provide a robust review that captured varying patient populations.

**Figure 1:**
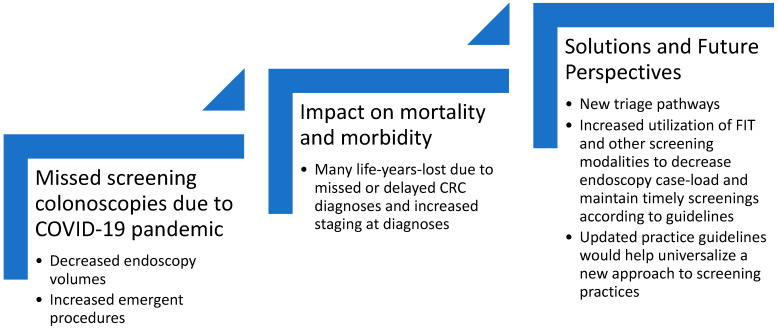
Overview of Impact of COVID-19 on CRC screenings.

Since the onset of the COVID-19 pandemic, new systems have been put in place to gather vast data as variants arise, efficacious vaccines are available and we have gained more knowledge about COVID-19, including the risk of infection in different medical settings. It is important to make up for the backlog of cancer screenings and subsequent deficit of diagnoses in 2020 [8,9,10,11]. Colorectal cancer is the third most common cancer and second leading cause of cancer-related death in the United States [12]. There have been an estimated nearly 4 million missed colorectal screening examinations due to COVID-19 [9]. Discernibly, a lack of screenings will result in late or missed cancer diagnoses for many patients. This review highlights trends in colorectal cancer screening, the potential effects on the morbidity and mortality of colorectal cancer, and proposed solutions to overcome the negatives effects of missed cancer screenings. (Figure 1 provides a summary of the key aspects covered by this review).

## 2. Impact of COVID-19 on Colorectal Cancer Screening

### 2.1. Decrease in CRC Screening Procedures

Early in the pandemic, multiple GI and cancer societies put forth recommendations to postpone non-urgent procedures cancer screenings to conserve healthcare resources and reduce the exposure of healthcare workers to COVID-19 [13,14,15]. This guidance led to a significant decline in the number of new cancers identified during the pandemic internationally. (A summary of all retrospective studies on endoscopy volume and impact on CRC detection rates is found in Table 1) In the US, Kaufman et al. reported that there was a 46.4% decline in the number of cancers identified weekly among breast, colorectal, lung, pancreatic, gastric, and esophageal cancers from March to April 2020 [16]. London et al. performed an analysis showing a decrease in colorectal cancer screening by 84.5% from January of April of 2020 compared to the previous year. This translated to a 39.9% reduction in malignant CRC diagnoses [17]. Multiple retrospective studies at pathology sites in Italy report decreased CRC diagnosis rates ranging from 46–62%, the largest decline compared to other cancer diagnoses over a 10- week period during the first countrywide lockdown [18,19]. In the UK, Rutter et al. determined that from the end of March to the end of May 2020, at the first peak of COVID-19, there was a decrease of endoscopic procedures to a mere 12% of the pre-COVID volume and weekly cancer detection rates decreased by 58% over all cancers, 72% of which were missing colorectal cancer diagnoses [20]. These drastic rates of decline in endoscopy volumes correlate with a global survey of endoscopy units from 55 countries that reported an average of 85% decreased volume [21].

Morris et al. followed UK endoscopy trends through October 2020 and highlighted an important detail. There were over 60% fewer two week wait referrals for colonoscopies in high-risk patients with suspected CRC in April 2020 compared to 2019, but these numbers recovered to 2019 averages by October 2020. However, this resulted in a backlog of an estimated 3500 missed CRC diagnoses and treatments between April and October 2020 [22]. It can generally be agreed that the initial changes in procedural practices for non-urgent concerns was appropriate given the gravity of COVID-19 pandemic. However, the decrease in CRC screening endoscopies worldwide has important implications that will be devastatingly long-lasting if proper steps are not taken to address possible missed cases.

### 2.2. Impact on Severity and Stage of CRC Diagnoses

Moreover, there is also consistent evidence of higher rates of cancer-related emergencies and more advanced stages at detection due to COVID-19. A single center study at a tertiary care center in the UK found that over the entire year, 2020 saw a significant increase in patients presenting with emergent large bowel obstructions and T4 cancers at diagnosis than both 2018 and 2019 [23]. Mizuno et al. describe greater rates of emergent CRC cases due to complete large bowel obstructions in a cohort of patients who underwent surgery in a cancer treatment center in Japan during the first state of emergency [24]. In Spain, a single center study in Navarra reported a significant increase in CRC diagnoses in emergency setting during the first surge of the pandemic. Fewer diagnoses were made through the screening program and more patients were diagnosed with metastatic disease at diagnosis compared to the same period in 2019 [25]. Similar trends with more advanced disease at diagnosis was observed in Brazil [26]. (Studies summarized in Table 2)

### 2.3. Models Predicting Morbidity and Mortality Impact

With such significant reductions in screening and treatment, there is a reasonable expectation and concern that there will be an increase in CRC attributable deaths. Several models have been developed to estimate the impact of delayed screening on cancer deaths. According to a UK-based study by Maringe et al., compared to pre-pandemic figures, there will be an estimated increase of 1445–1563 additional deaths (15.2–16.6% increase) over five years as a result of the delayed diagnoses caused by the COVID-19 pandemic [27]. In a similar study, it was estimated that without implementing “catch-up” screening protocols, a 12-month disruption in screening will result in an expected increase of 1360–3968 additional deaths across the Netherlands, Australia, and Canada compared to no disruption in regular screenings [28].

In an Australian study, it was estimated that a six-month delay in colorectal cancer screening would result in an increase of over $1.2 million in healthcare costs [29]. Models and predictions are summarized in Table 2.

### 2.4. Triage and Alternate Screening Pathways

To address the growing backlog of cases seen at every endoscopy unit, multiple physician groups proposed adept triage pathways to identify high risk patients and prioritize alternate screening options like fecal immunochemical testing (FIT) for lower risk groups [30]. Studies of screening triage pathways are summarized in Table 1. Most studies that implemented these new pathways were completed in the UK in an effort to reduce the number of patients on the two week wait referral list for colonoscopy. In one study, Maclean et al. offered all referred patients a FIT test. 94% of samples were returned, of which 34% were interpreted and positive and were followed by colonoscopy. The CRC diagnosis rate with this pathway was 3.7% (14 CRC of 122 colonoscopies), comparable to the pre-COVID detection rate of 3.9% [31]. Miller et al. triaged referred patients to alternate testing modalities of FIT with CT scan, FIT only, direct colonoscopy or clinical follow up. 98% of patients were triaged to the FIT+CT or FIT groups and were followed up with colonoscopy if deemed appropriate based on results. Using this pathway, they achieved a CRC detection rate of 3.1%, which is clinically similar to the CRC detection rate from 2017–2019 (3.3%) [32]. Another study utilized only clinical evaluation, CT scan or direct colonoscopy as options for triaging referred patients, which also yielded an overall similar CRC detection rate to pre-pandemic rates [33].

## 3. Conclusions

COVID-19 has disrupted many aspects of daily life and has transformed how almost every industry functions. The practice of medicine and how and when we deliver care to patients is no exception. COVID-19 surges and lock-downs delayed colorectal cancer screening on many patients and while endoscopy units are slowly returning to pre-pandemic practices, the missed procedures must be addressed. Without utilizing evidence based tests and re-working screening algorithms, like the triage pathways discussed here, there will be a significant economic and social impact on quality of life and life years lost. Fortunately, we have multiple tools in our armory to ensure that we continue to provide patients with appropriate preventative care. It is important that we begin to consider these options as we continue to navigate this pandemic. Triaging low versus high risk patients and using FITs (both quantitive lab-read and point-of-care) allows timely screening and focuses the work-load of endscopy units on the highest risk patients. This is especially important because resistent strains and new surges remain a real threat in prolonging this pandemic. It would be prudent to establish practice guidelines that employ multiple clinical screening methods to risk-stratify patients, diagnose colorectal cancer early and treat it appropriately as we attempt to return to on-time screening of all eligible patients and catch up with the pandemic-driven missed screenings. The goal must be to catch missed diagnoses and reach the same detection, treatment and cure rates as before the COVID-19 pandemic, with as little delay as possible. The recent emergence of the Delta and other variants of COVID-19 has undoubtedly complicated matters, which will only serve to delay this goal.

## Figures and Tables

**Table 1:** Summary of studies reporting impact of COVID-19 on endoscopy volumes and CRC rates.

Retrospective Studies on Impact on Endoscopy Volumes and CRC Detection				
First Author	Year	Country	[% Decrease] in [Procedure] during [Time Frame]	Summary Point
Kruse-Diehr AJ	2021	US	90% decrease in colonoscopies and biopsies from April 2019 to April 2020	single site data; FIT testing and more screening help the backlog of cases
London JW	2020	US	84.5% decrease in CRC screening from 2019 to 2020	possible stage increase due to less screening
Rutter MD	2021	UK	89.7% decrease in colonoscopies from January–March 2020 to March to May 2020. The number of CRC detected in that same timeframe decreased by 71.1%	significantly fewer CRC diagnoses and the delay will incur a significant impact on survival
Morris EJA	2021	UK	During peak COVID-19, there was a 63% reduction in referrals and a 92% reduction in colonoscopies. The pre-COVID rate returned 6 months later.	new waves of COVID-19 will continue to cause decrease in screenings; solutions are needed
Miller J	2021	UK		proposed solution to case backlog: priority to suspected cancer patients and gives them treatment based on this
Shinkwin M	2021	UK	There was a significant decrease in referrals and colonoscopies and increased number of emergencies	pathway to treatment must improve in order to solve the problem
Barrett K	2020	UK	50% reduction in screening endoscopies from 2020 to 2019	a combination of a consultant-led phone triage system with FIT could be the best way to solve backlog
Maclean W	2020	UK	28% decrease in colonoscopies from October–December 2019 to March–July 2020	use of FIT would optimize the use of resources by triaging pts in order of need for colonoscopy
Suárez J	2021	Spain	New diagnoses of CRC decreased 48% from March–June 2019 to March–June 2020 and higher amount diagnosed in emergency settings	decreased number of CRC screenings and diagnoses, leading to increased incidence of emergent presentations of CRC in the ER
Mizuno R	2020	Japan	The number of colonoscopies was decreased during the pandemic leading to an increased incidence of obstructive CRC	screening systems should be reorganized for future pandemics
Ferrara G	2021	Italy	CRC diagnosis fell by 46.6% and overall cancer diagnosis fell by 44.9%	if colonoscopy is indicated by FIT result but wait times are too long, consider triaging pts w/CT colonography or double contrast barium enema
De Vincentiis L	2021	Italy	CRC diagnoses decreased 62% from 2018+19 to 2020	triage pts with FIT; in the case of excessive wait for colonoscopy, consider CT colonography or double barium enema
Aguiar S	2021	Brazil	CRC diagnosis decreased significantly, significant increase in locally diagnosed patients	there was a financial barrier in the diagnosis of patients

Abbreviations: CRC: colorectal cancer; ER: emergency department; FIT: fecal immunochemical test.

**Table 2:** Summary of models and studies’ prediction impact on morbidity and mortality of delayed screening and alternate screening pathways.

Model Studies for Impact of Delayed Diagnosis/Reduced Screening and Proposed Solutions/Time to Catch up with Backlog						
First Author	Year	Country	[%] Increase Mortality/Loss of Life Years] over [n months] Time Delay	[%] Stage Shift to [stage] Due to [n months] Time Delay	Estimated Time to Recover Backlogged Case	Proposed Solution/Summary Point
Sud A	2020	UK	3 month delay with 25 %, 50%, and 75% backlog model = 90 lives lost/1662 life years, 183 lives lost/3362 life years, and 276 lives lost/5075 life years, respectively. About 7 life years lost per patient			Models show significant increases in mortality and life-years lost due to treatment delay
Loveday C	2020	UK	2/4/6 month delay resulted in 653/1419/2250 lives lost and 9214/20 315/32 799 life years lost.			FIT testing could help solve the backlog of cases and reduce the amount of lives lost
Sud A	2020	UK	3/6 month delay results in 4755/10760 deaths and 92214/208275 life years lost in all cancers			Delays in screening will have significant impact on survival, so to avoid more public health crises it is necessary to address the backlog
Maringe C	2020	UK	15.3–16.6% increase in deaths over 5 years due to delays			Delays in screening will lead to a large number of additional deaths
Kregting LM	2021	Netherlands	0.0025% increase in death from CRC over 10 years without catch-up	3% shift from stage I to stage II with a 6 month delay.		The best way to minimize mortality and cancer incidence is to catch up on missed screenings
Dinmohamed AG	2020	Netherlands				Less CRC have been diagnosed during COVID and the effects of this are not yet clear
de Jonge L	2021	Netherlands, Canada, Australia	3/6/12 month delay would cause an increase of 0.2–0.3%/0.4–0.6%/0.8–1.2% of deaths in the next 30 years without catch-up screening in the Netherlands. 3/6/12 month delay would cause an increase of 0.5%/1.0%/2.0% of deaths in the next 30 years without catch-up screening in Australia. A 12 month delay would cause an increase of 0.8% of deaths in the next 30 years without catch-up screening in Canada			Catch-up screening is necessary as soon as possible in order to avoid unnecessary CRC burden
Lui TKL	2020	China		6.4% of pts with CRC would present with stage increases with 6 month delay		There has been a substantial drop in endoscopies and endoscopy services must be resumed as soon as possible
Ricciardiello L	2020	Italy	12% increase in mortality with 12 month delay	4–6 month delay leads to 2% shift from stage I to stage II		Reorganize efforts against high impact diseases
Yong JH	2020	Canada	3/6/12 month delay could lead to 110/250/960 additional deaths	1100 or 2200 more colorectal cancer cases respectively, with over 60% of the cases at an advanced stage (III or IV)		Delays in screening will cause large adverse effects on long-term cancer outcomes
Degeling K	2021	Australia	3 excess deaths over 5 years with a 3 month delay. 11 excess deaths over 5 years with a 6 month delay.	3% shift from stage I to stage II with a 3 month delay. 5.9% shift from stage I to stage II with a 6 month delay.		3 and 6 month delays in cancer screenings are projected to lead to hundreds of life-years lost and a significant medical impact
Ng J	2021	US	A 2/6 month delay caused a 4.8%/21.3% increase in tumor control risk and a 5%/6% increase in distal metastasis risk			Treatment delays will lead to worse cancer outcomes and It is necessary to develop quantitative models to guide approaches to solutions
Tinmouth J	2020	Canada			41 months w/o redirection of pts to FIT or 28, 22, 19 months with redirecting 25%, 50%, or 75% to FIT	Redirect low-risk colonoscopies to FIT testing or increase hospital capacity for endoscopies

Abbreviations: CRC: colorectal cancer; FIT: fecal immunochemical test.

## Data Availability

Not applicable.
